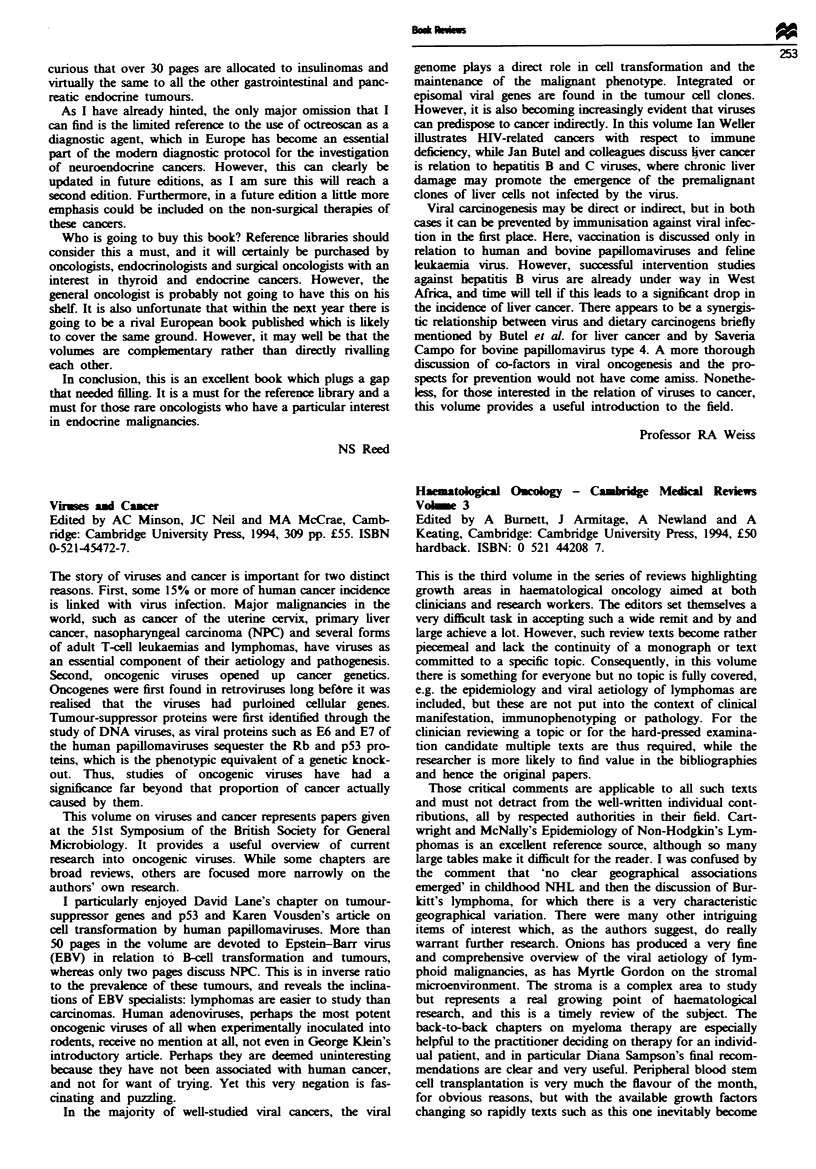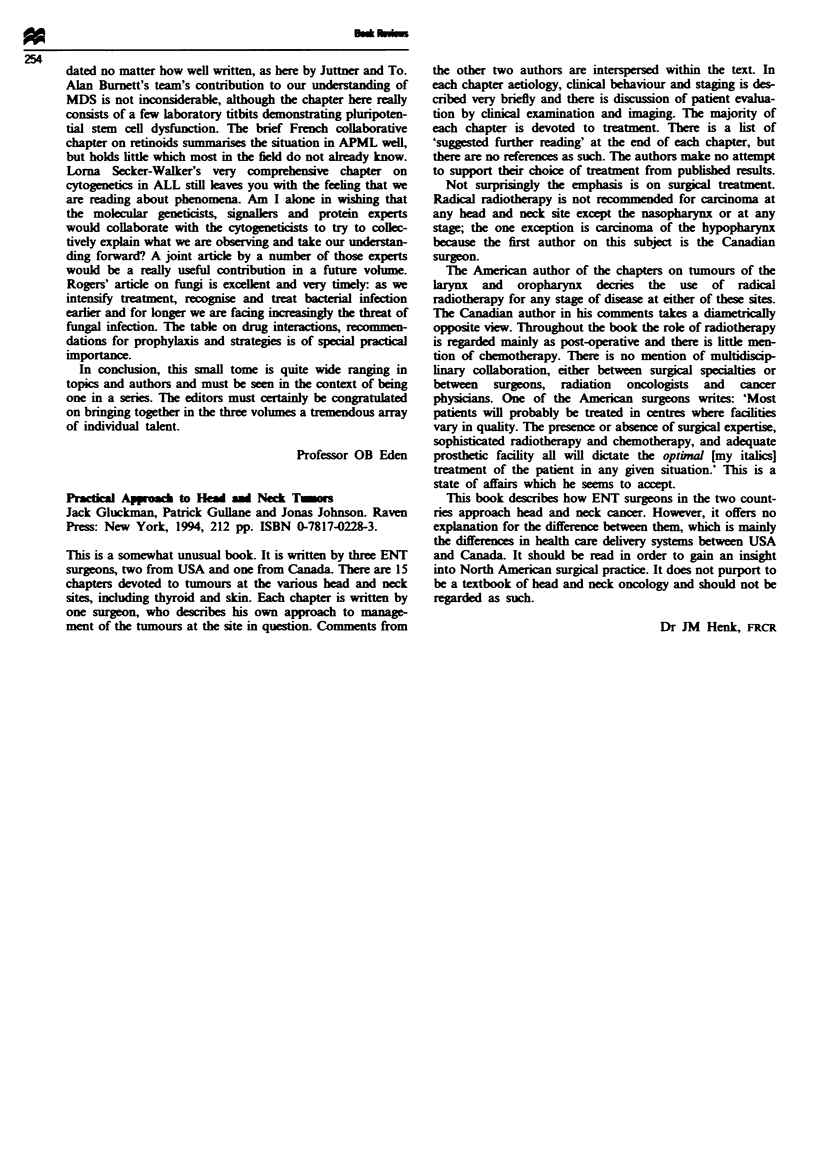# Haematological Oncology - Cambridge Medical Reviews Volume 3

**Published:** 1995-07

**Authors:** OB Eden


**Haematological Oncology – Cambridge Medical Reviews Volume 3**

Edited by A Burnett, J Armitage, A Newland and A Keating, Cambridge: Cambridge University Press, 1994, £50 hardback. ISBN: 0 521 44208 7.

This is the third volume in the series of reviews highlighting growth areas in haematological oncology aimed at both clinicians and research workers. The editors set themselves a very difficult task in accepting such a wide remit and by and large achieve a lot. However, such review texts become rather piecemeal and lack the continuity of a monograph or text committed to a specific topic. Consequently, in this volume there is something for everyone but no topic is fully covered, e.g. the epidemiology and viral aetiology of lymphomas are included, but these are not put into the context of clinical manifestation, immunophenotyping or pathology. For the clinician reviewing a topic or for the hard-pressed examination candidate multiple texts are thus required, while the researcher is more likely to find value in the bibliographies and hence the original papers.

Those critical comments are applicable to all such texts and must not detract from the well-written individual contributions, all by respected authorities in their field. Cartwright and McNally's Epidemiology of Non-Hodgkin's Lymphomas is an excellent reference source, although so many large tables make it difficult for the reader. I was confused by the comment that 'no clear geographical associations emerged' in childhood NHL and then the discussion of Burkitt's lymphoma, for which there is a very characteristic geographical variation. There were many other intriguing items of interest which, as the authors suggest, do really warrant further research. Onions has produced a very fine and comprehensive overview of the viral aetiology of lymphoid malignancies, as has Myrtle Gordon on the stromal microenvironment. The stroma is a complex area to study but represents a real growing point of haematological research, and this is a timely review of the subject. The back-to-back chapters on myeloma therapy are especially helpful to the practitioner deciding on therapy for an individual patient, and in particular Diana Sampson's final recommendations are clear and very useful. Peripheral blood stem cell transplantation is very much the flavour of the month, for obvious reasons, but with the available growth factors changing so rapidly texts such as this one inevitably become dated no matter how well written, as here by Juttner and To. Alan Burnett's team's contribution to our understanding of MDS is not inconsiderable, although the chapter here really consists of a few laboratory titbits demonstrating pluripotential stem cell dysfunction. The brief French collaborative chapter on retinoids summarises the situation in APML well, but holds little which most in the field do not already know. Lorna Secker-Walker's very comprehensive chapter on cytogenetics in ALL still leaves you with the feeling that we are reading about phenomena. Am I alone in wishing that the molecular geneticists, signallers and protein experts would collaborate with the cytogeneticists to try to collectively explain what we are observing and take our understanding forward? A joint article by a number of those experts would be a really useful contribution in a future volume. Rogers' article on fungi is excellent and very timely: as we intensify treatment, recognise and treat bacterial infection earlier and for longer we are facing increasingly the threat of fungal infection. The table on drug interactions, recommendations for prophylaxis and strategies is of special practical importance.

In conclusion, this small tome is quite wide ranging in topics and authors and must be seen in the context of being one in a series. The editors must certainly be congratulated on bringing together in the three volumes a tremendous array of individual talent.

Professor OB Eden